# Choked to Death

**Published:** 1870-11

**Authors:** 


### CHOKED TO DEATH.

All that we eat or drink passes over the top of the open windpipe, without a particle ever entering it, although that opening is larger than a dime, because the very effort of swallowing draws over the open top a fleshy trap-door, which fits so closely, that not even a particle of air can pass; but the instant what is swallowed has passed over the trap-door downwards, it opens up with a spring, and we go on breathing as if nothing had ever happened; but if we attempt to swallow anything too large, this trap-door being at the narrowest part of the passage, is kept closed, not a particle of air can enter the lungs, and we die in a moment of suffocation, as in drowning or smothering.

If you chew a piece of dried beef for some time, there will be a white remnant left which there is no inclination to swallow; if it be taken and picked apart, it will appear to be made of little strings, tough and strong; these were attached to the more flesh-like parts, which were chewed and swallowed. If, in eating, a man has a sharp knife, and cuts his meat wholly in two, he may put two or three of these pieces in his mouth, and chew and swallow without danger; but if the knife is dull, and does not divide the piece wholly, two pieces may be tied together with one of these little strings, and while you have swallowed one part nearest the swallow, the other part may be near the teeth, and both held by the string, which, holding the two parts together, and hanging across the trap-door, prevents its opening, and death follows in an instant; hence the practical value of sharp knives at the dinner table. A long hair in a mouthful of food may so entangle it in the act of swallowing, as to cause a choking to death; this is what is meant by being "strangled by a hair." "String beans" may occasion a choking to death in the same way, if not carefully "strung." Hence, all food should be cut fine; should be thoroughly divided with a sharp knife; should be taken into the mouth in small pieces, chewed thoroughly, and swallowed deliberately.

Most readers have suffered considerable inconvenience from something "going the wrong way;" this is occasioned by a single drop of water, or atom of solid food, a crumb or other thing slipping into the windpipe, and falling down to the lungs, causing an instantaneous, spiteful, angry, dry cough; it is because nature was alarmed by an unnatural and unwelcome visitor, and takes this, her only means of ejecting the intruder. If the particle is large or heavy, the surgeon must be called to cut open the windpipe and remove the substance.

A person cannot laugh or speak a word, unless the top of the windpipe is uncovered; but if a laugh is provoked, or a word attempted to be spoken while in the act of swallowing, and just before the particle has fully passed over the trap-door, it is raised a little, a drop or crumb falls into it, and hence the mischief. Hence in eating, do not attempt to speak until the "swallow" is clear.

